# Risk Factors for One-Year Mortality in Hospitalized Adults with Severe COVID-19

**DOI:** 10.14336/AD.2022.0424

**Published:** 2023-02-01

**Authors:** Rodrigo Núñez-Cortés, Rubén López-Bueno, Rodrigo Torres-Castro, Camilo Soto-Carmona, Maritza Ortega-Palavecinos, Sofía Pérez-Alenda, Lilian Solis-Navarro, Óscar Díaz-Cambronero, Francisco M. Martinez-Arnau, Joaquín Calatayud

**Affiliations:** ^1^Department of Physiotherapy, Physiotherapy in Motion Multispeciality Research Group (PTinMOTION), University of Valencia, Valencia, Spain.; ^2^Departament of Physical Therapy, Faculty of Medicine, University of Chile, Santiago, Chile.; ^3^Institut d’Investigacions Biomèdiques August Pi i Sunyer (IDIBAPS), Barcelona, Spain.; ^4^Hospital Clínico Dra. Eloisa Díaz de La Florida, Santiago, Chile.; ^5^Department of Physical Medicine and Nursing, University of Zaragoza, Spain.; ^6^National Research Centre for the Working Environment, Copenhagen, Denmark.; ^7^International Physiotherapy Research Network (PhysioEvidence), Barcelona, Spain.; ^8^Department Anesthesiology, Hospital Universitari i Politécnic la Fe, Valencia, Spain.; ^9^Perioperative Medicine Research Group. Biomedical Research Institute la Fe, Valencia, Spain.; ^10^Exercise Intervention for Health Research Group (EXINH-RG), Department of Physiotherapy, University of Valencia, Spain.

**Keywords:** aging, SARS-CoV-2, coronavirus, mortality, infectious diseases

## Abstract

As the body's immunity declines with age, elderly-hospitalized patients due to COVID-19 might be at higher mortality risk. Therefore, the aim of this prospective study was to examine the possible risk factors (demographic, social or comorbidities) most associated with mortality one-year after diagnosis of COVID-19. Routine data were collected from a cohort of hospitalized adults with severe COVID-19. The primary endpoint was mortality at one-year after diagnosis of COVID-19. We used a Cox proportional hazard model to estimate the hazard ratios (HRs) for both all-cause and specific cardiorespiratory mortality. A fully adjusted model included sex, socioeconomic status, institutionalization status, disability, smoking habit, and comorbidities as confounders. A total of 368 severe cases hospitalized on average 67.3 ± 15.9 years old were included. Participants aged ≥ 71 years had significantly higher HRs for all-cause mortality (adjusted HRs = 2.86, 95%CI: 2.01-4.07) and cardiorespiratory mortality (adjusted HRs = 2.86, 95%CI: 1.99-4.12). The association between age and mortality after diagnosis of COVID-19 due to both all-causes and cardiorespiratory mortality showed a consistent dose-response fashion. Institutionalization, disability, and socioeconomic status also showed a significant association with mortality. In conclusion, aging itself was the most important risk factor associated with mortality one year after diagnosis of COVID-19. People with disabilities, institutionalized or low socioeconomic status are significantly more likely to die after COVID-19.

Although the current COVID-19 mass immunization campaign has generated a change in the course of the pandemic, the risk of impact of new circulating variants (e.g. Omicron variant, Delta), should not be underestimated at all [1]. Severe acute respiratory syndrome SARS-CoV-2 continues to affect more than 500 million people worldwide and to cause more than 6 million deaths [2, 3]. Approximately 20% of infected cases require hospitalization and 6% require admission to intensive care units (ICUs) [4]. These groups include especially people with comorbidities and older adults [5, 6].

## Age and other risk factors

Aging is a complex process associated with several changes at the organismal, tissue, cellular and molecular levels [7]. This phenomenon leads to increased susceptibility to infectious diseases and contributes to the development of cardiovascular, metabolic, autoimmune and neurodegenerative diseases [8, 9]. Additionally, the immune system is itself also influenced by age-associated changes occurring in such physiological systems as the endocrine, nervous, digestive, cardio-vascular and muscle-skeletal systems [7].

Older adults are more likely to die from COVID-19, as the body's immunity declines with age [10]. In addition, COVID-19 in older adults is often present with atypical symptoms, such as confusion, acute mental changes, decreased mobility, poor appetite, incontinence, dysphagia, and tachypnoea, making it difficult to identify and treat early [11]. The physiological changes of aging are often accompanied by multiple chronic conditions that contribute to poor health and increased risk of death [12]. In particular, long-term care residents are one of the most at-risk groups due to their chronic disease burden and the impact of congregate housing [11, 12]. While there is a strong association between COVID-19 mortality and cardiometabolic comorbidities such as hypertension, obesity or diabetes [13], other factors have also been shown to be strongly associated with mortality. For example, people with disabilities and people with low socioeconomic status are also a risk group [14, 15], and there is an urgent need to improve health care for these patients.

## Consequences of hospitalization for COVID-19

Patients who survive hospitalization for COVID-19 have a high prevalence of physical sequelae (i.e., limited physical capacity) [16], loss of independence in activities of daily living [17], persistence of respiratory symptoms [18] and impaired pulmonary function [19], being more susceptible to develop health-related quality of life impairment [20]. Thus, worsening health can have serious long-term consequences and it is important to determine which risk factors lead to an increased risk of mortality after COVID-19 diagnosis. Despite this, to date most of the studies have focused on the short-term effects of COVID-19. For example, there is considerable evidence of mortality from COVID-19 in the hospital environment [21]. However, little is known about COVID-19 mortality after hospital discharge. We know that frailty is a risk factor for mortality in older adults [22], and we also know that COVID-19 generates sequelae on multiple systems, but especially respiratory, cardiovascular and physical [23, 24]. Thus, it is important to know what happens with the older adults after long-term follow-up. Specifically examining the multiple factors that determine poor prognosis is essential to develop control strategies and early interventions. Therefore, the aim of this study was to examine the possible risk factors (demographic, social or comorbidities) most associated with mortality one-year after diagnosis of COVID-19.

## Methods

In this prospective study, routine data from a cohort of patients with severe COVID-19 were collected at the Hospital Clínico La Florida (Santiago, Chile) between April and June 2020, during the first wave of the COVID-19 pandemic. This institution corresponds to a public health center located in an urban area composed mainly of middle socioeconomic families [25]. Inclusion criteria were the following: age ≥ 18 years; diagnosis of COVID-19 by reverse transcription-polymerase chain reaction (PCR) [4]; severe cases admitted to the hospital for respiratory failure requiring vasoactive medication, oxygen therapy and/or mechanical ventilation [26]. There were no exclusion criteria, since data were collected from all severe cases hospitalized during that period of time. The study was approved by the Ethics Committee of the Southeast Metropolitan Health Service (Santiago, Chile).

## Risk factors

Sociodemographic data and underlying comorbidities were collected from each patient's electronic medical records during hospitalization, between April and June 2020. Data collected included: I) Sociodemographic data (age and sex); II) Clinical data: date of diagnosis, date of hospital discharge, ICU admission history; III) Institutionalization status (yes/no); IV) Pre-existing comorbidities recorded during hospital admission, such as obesity, hypertension, diabetes, cardiovascular disease, cerebrovascular disease, chronic respiratory disease, chronic kidney disease or cancer; V) Income was used as an indicator of socioeconomic status. Socioeconomic status was categorized according to the individual monthly taxable income that determines the level of benefits for the Chilean public health system guarantees (i.e., equal to or less than US$320); VI) Smoking status (yes/no) was defined as active smoker or with a history of smoking versus non-smoker; VII) Disability status was defined according to the hospital's social register, which classified as persons with disabilities those with long-term physical, mental, intellectual or sensory impairments. All data were collected by three researchers from the institution (RNC, CSC, MOP).

## Outcome

The primary endpoint was mortality at one-year after diagnosis of COVID-19. All-cause mortality or specific cardiorespiratory mortality was defined according to the death certificate of the National Oficial Registry, as well as the date of death. The follow-up time was up to June 30, 2021, or the date of death.

**Table 1:** Characterization of total sample.

Subjects (n)	368
Age <71 year ≥ 71 year	183 (49.7%) 185 (50.3%)
Sex Male Female	208 (56.5%) 160 (43.5%)
Socioeconomic status Low High	275 (74.7%) 93 (25.3%)
Hospital stay (days)	13 (1-82)
Admission to ICU	70 (19.0%)
Death from cardiorespiratory causes	170 (46.2%)
Death from all causes	178 (48.4%)
Smoking history	28 (7.6%)
Person with disability	52 (14.1%)
Institutionalized adults	28 (7.6%)
Comorbidities Obesity Hypertension Diabetes Cardiovascular disease Cerebrovascular disease Chronic respiratory disease Chronic kidney disease Cancer	67 (18.2%) 246 (66.8%) 155 (42.1%) 69 (18.8%) 65 (17.7%) 70 (19%) 41 (11.1%) 21 (5.7%)

Values are presented in frequencies (%) unless otherwise indicated. Abbreviations: ICU, intensive care unit.

## Statistics

We conducted all statistical analyses in Stata version 16.1 (StataCorp, Texas, USA). We used a Cox proportional hazard model to estimate the hazard ratios (HRs) for both all-cause and specific cardiorespiratory mortality. Time-on-study in days was used as the timescale. Age was categorized into two groups using the median to set the cut-off point and served as exposure variable, and the lower category of age was set as reference. Two models were tested; a model adjusted for age and sex (model A) and a fully adjusted model (model B) that included age, sex, socioeconomic level, institutionalization status, disability, smoking habit, and comorbidity as confounders. The proportional hazards assumption was checked by testing interactions with log(time) using stphplot command, finding no evidence of assumption violation. After assessing interactions using a chunk test between full and reduced models, no significant interactions were found. The results were displayed as forest plots.

Due to the strong association between age and COVID-19 mortality, we also assessed the dose-response associations of age (modelled as a continuous exposure) and both all-cause and cardiorespiratory mortality using a restricted cubic spline model to allow for potential non-linearity. We trimmed observations less than 5% and greater than 95% of the distribution and pre-specified knots placed at the 10th, 50th, and 90th percentiles of the exposure distribution [27]. Linearity was assumed for values below the 10th percentile and for values above the 90th percentile. Results are reported as HRs with 95% confidence intervals (CIs) and levels of significance were set at p < 0.05.

## Results

**Figure 1.**
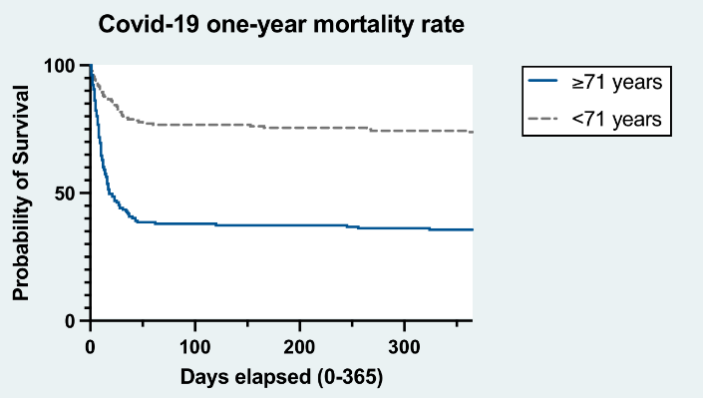
Prospective associations between age and mortality among hospitalized patients due to COVID-19.

A total of 368 severe cases hospitalized for COVID-19 were included, which corresponded to 32% of the total number of patients diagnosed with COVID-19 in the hospital during the recruitment period. There was no loss to follow-up and all cases were analyzed. Table 1 describes the basic characteristics of the study sample. The mean age was 67.3 ± 15.9 years. The total number of deaths during the one-year follow-up was 178 cases. Mortality due to cardiorespiratory causes was 26.9% in patients aged <71 years and 65.1% in those aged ≥71 years. All-cause mortality was 28.6% in patients aged <71 years and 67.7% in those aged ≥71 years. Seventy-nine percent of the patients died in hospital, while 21% died after hospital discharge. The high mortality risk was observed mainly in the first three months after diagnosis of COVID-19 (Fig. 1).

Participants aged ≥ 71 years had a significantly higher HRs for all-cause mortality (crude HRs = 3.45 [95%CI: 2.47-4.81]; adjusted HRs = 2.86 [95%CI: 2.01-4.07]) and cardiorespiratory mortality (crude HRs = 3.46 [95%CI: 2.48-4.87]; adjusted HRs = 2.86 [95%CI: 1.99-4.12]) compared with their younger counterparts. Socioeconomic level, institutionalization status and disability also showed a significant association with mortality (p<0.05).

**Figure 2.**
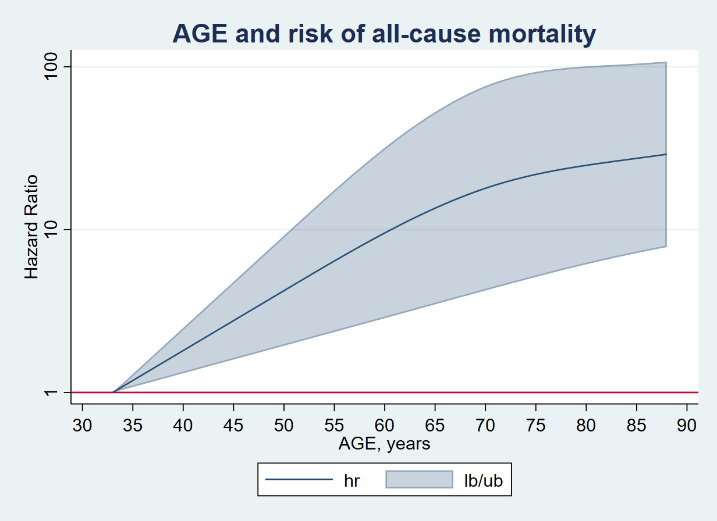
Dose-response association (Adjusteda hazard ratios and associated 95% confidence interval band) between continuous age index and all-cause mortality in hospitalized patients due to COVID-19. aAdjusted for Model B (Full) including sex socioeconomic level, institutionalization status, disability, smoking habit, and comorbidity. Dose-response associations were assessed with restricted cubic splines with knots at 10th, 50th, and 90th centiles of the distribution of the exposure of interest (reference = Lowest value). Abbreviations: hr= hazard ratio lb/ub=lower boundary/upper boundary

**Figure 3.**
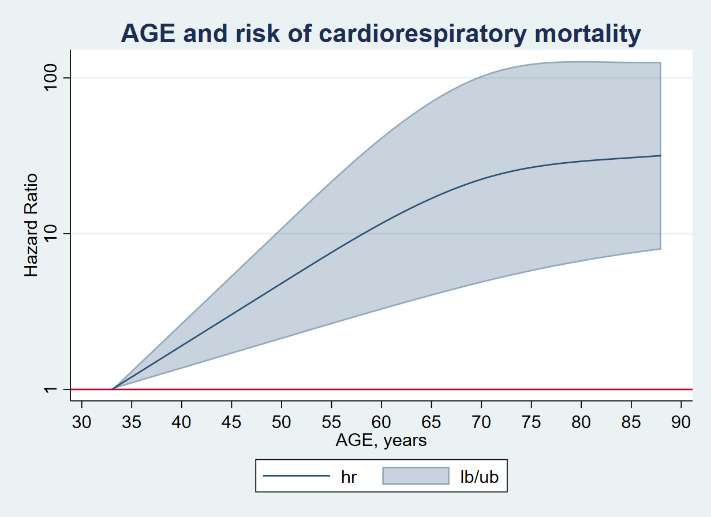
Dose-response association (Adjusted^a^ hazard ratios and associated 95% confidence interval band) between continuous age index and cardiorespiratory mortality in hospitalized patients due to COVID-19. ^a^Adjusted for Model B (Full) including sex socioeconomic level, institutionalization status, disability, smoking habit and comorbidity. Dose-response associations were assessed with restricted cubic splines with knots at 10th, 50th, and 90th centiles of the distribution of the exposure of interest (reference = Lowest value). Abbreviations: hr= hazard ratio lb/ub=lower boundary/upper boundary

Figures 2 and 3 show the dose-response association between adjusted continuous age and all-cause and cardiorespiratory mortality respectively. A consistent steadily and substantial increase of HRs was observed overall in both the crude and the adjusted models, although a slight flatten of the curve was also observed over the age of 65 years.

## Discussion

Our study examined demographic, social, or comorbidity risk factors associated with mortality one-year after COVID-19 diagnosis in a cohort of severe inpatient cases during the first wave of the pandemic. The main finding of this study was the significant association between older age and a higher probability of COVID-19 mortality at one-year after diagnosis of COVID-19. Although older age was the major risk factor, socioeconomic level/status, institutionalization status and disability also played an important role, attenuating the examined association.

These results are consistent with two previous meta-analyses that have reported an association between older age and an increased risk of mortality [10, 28]. It is important to emphasize that these meta-analyses explored in-hospital mortality and not in a follow-up as our data. It seems logical that age itself is the most significant risk factor for death from COVID-19, since this population has more chronic conditions that increase the risk of death [29]. However, our study suggests that older age was a risk factor for mortality independently of chronic conditions. Therefore, these results could be better explained by age-related decline and dysregulation of immune function [30]. Although aging-related impairment of the immune response has not been investigated in detail in COVID-19, it can be inferred to be the main reason for the increased susceptibility to infections in older adults for several reasons. For example, Kadambari et al. [31] and Moss et al. [32] proposed that pre-existing cytomegalovirus infection (which approaches 80% in older) is associated with the acceleration of immune senescence and may promote increased inflammation-mediating cytokines, such as IL-6, worsening the clinical outcome of COVID-19 [31]. On the other hand, the type 1 interferon (IFN) response is weaker in older adults and is suppressed against SARS-CoV-2, leading to a poor CD8+ T cell response to viral infection and high levels of pro-inflammatory cytokines [12, 30, 33]. Thus, inflammation and cytokine storm may lead to immunopathology and severe disease [30]. In addition, regulatory T cells (Tregs) are an important subpopulation of T cells that exert immunosuppressive effects, and these are significantly reduced in patients with COVID-19 [34]. The higher mortality rate in older adults with comorbidity could also be related to the decrease in Treg levels. This suggests that alternative immunotherapeutic strategies for this disease are necessary [34].

While these findings indicate that aging itself is an important risk factor for death from COVID-19, other aspects might also be important. For example, our results reinforce those reported by Ramirez and Lee in a cross-sectional study [14], indicating that social determinants, such as poverty, were significantly and positively associated with a higher COVID-19 mortality rate in older adults. These results are not surprising, as pandemics historically cause higher mortality rates in populations with higher vulnerability (e.g., the 1918-1919 influenza pandemic) [35]. Health-care professionals should be aware of inequalities caused by socioeconomic gaps and address the excess and long-term mortality of this particular group.

Interestingly, people with disabilities were also shown to be a group at increased risk of mortality. In general, disabled people are more likely than non-disabled people to suffer from poverty and difficulty accessing medical care [36, 37]. Our findings are consistent with those reported by Bosworth et al. [38] who found in a retrospective study that those patients with self-reported disability were also at excess risk for all causes of death during the first two waves of the COVID-19 pandemic. These results imply the need to improve access to health care in this at-risk group, and to address the gaps leading to excess mortality, both during and after the COVID-19 pandemic.

A recent meta-analysis found that the pooled mortality among patients with critical or severe COVID-19 was 20.48% [39]. In contrast, the mortality risk in our COVID-19 patients was generally higher (48%). This could be explained by the fact that, although we included a cohort of cases that developed the severe form of the disease (i.e., hospitalized patients requiring vasoactive medication, oxygen therapy or mechanical ventilation), in general, the mean age was higher in our group and 75% had a low socioeconomic level. On the other hand, there were practically no differences in all-cause mortality and cardiorespiratory mortality. The few COVID-19 patients who died of causes other than cardiorespiratory-specific death had a diagnosis of cancer. In this context, a recent meta-analysis concluded that cancer patients were more susceptible to death from COVID-19 [40]. The high mortality rate in patients with cancer and COVID-19 is associated with both general risk factors and risk factors unique to cancer patients [41].

A strength of this study is the large sample size and the long-term follow-up of the patients. To date, few studies have presented follow-up data on mortality one year after COVID-19 and these have evaluated specific risk factors or specific populations. For example, a recent meta-analysis [42] evaluating all-cause mortality after discharge in patients recovered from COVID-19 only found four studies with one-year follow-up [43-46]. Pourhoseingholi et al. predicted one-year mortality after COVID-19 based on chest computed tomography [44], Roig-Marín et al. evaluated dementia as a risk factor for mortality in elderly patients admitted for COVID-19 [45], Maestre-Muñiz et al. described the causes of death per year in a sample of older adults [43], and Chai et al. evaluated risk factors related to one-year COVID-19 mortality in patients with cancer [46]. Therefore, to our knowledge, our study is the first to evaluate multiple risk factors for mortality one-year after diagnosis of COVID-19 in cases of hospitalization for severe disease. These results could contribute to the development of prevention and promotion strategies in public health, prioritizing resources in the most vulnerable groups to reduce the socio-health impact on future generations. In contrast, this study has some limitations. First, the possible underreporting of comorbidities in each patient's medical history could increase the possibility of residual confounding and underestimate the true strength of their association with mortality. Second, no data were collected regarding other variables that could influence patient mortality, such as physical inactivity, body mass index, frailty, or other indicators of socioeconomic status (e.g., educational level). Finally, it is important to note that these results only apply to the reality of one country and, therefore, multicenter research is required to confirm these results.

## Conclusions

Aging itself is the most important risk factor associated with mortality one-year after diagnosis of COVID-19. In addition, having disabilities, being institutionalized, or having low socioeconomic status significantly increase COVID-19 mortality risk.
